# Qualitative and Quantitative Evaluation of an Innovative Primary and Secondary Diabetes Clinic in Western Sydney

**DOI:** 10.5334/ijic.7548

**Published:** 2024-02-21

**Authors:** Sumathy Ravi, Gideon Meyerowitz-Katz, Anandhi Murugesan, Julie Ayre, Rajini Jayaballa, Duncan Rintoul, Marina Sarkis, Kirsten McCaffery, Glen Maberly, Carissa Bonner

**Affiliations:** 1 Western Sydney Diabetes, Integrated and Community Health, Western Sydney Local Health District, Blacktown, NSW, Australia; 2 Sydney Health Literacy Lab, Sydney School of Public Health, Faculty of Medicine and Health, The University of Sydney, Sydney, NSW, Australia; 3 School of Health and Society, University of Wollongong, Wollongong, NSW, Australia; 4 Rooftop Social, Bulli, NSW, Australia; 5 Agency for Clinical Innovation, St Leonards, NSW, Australia

**Keywords:** Type 2 diabetes, virtual care, telehealth, community clinic, evaluation, integrated care

## Abstract

**Introduction:**

Western Sydney Diabetes (WSD) established an innovative diabetes service in May 2020, using virtual and in-person care, linking primary care with the diabetes specialist team. This study evaluated the service’s feasibility using qualitative and quantitative methods.

**Method:**

Evaluation included: 1) thematic analysis of interviews and workshops with patients and health professionals (n = 28); 2) quantitative analysis of records of patients admitted July 2020–June 2021 (n = 110).

**Results:**

Key themes related to 1) benefits: convenient location, access to integrated care, advantages of virtual care; 2) challenges: hard for patients to ask questions, technology issues; 3) confidence: shared care decision making, multidisciplinary team; and 4) future directions: additional multidisciplinary services, expanded insulin stabilisation service, promotion.

Improvements between baseline and 3 months included 1.3% reduction in HbA_1c_ (p < 0.05). Sulfonylurea dropped by 25% between initial appointment and follow-up, and GLP1RA/SGLT2i use increasing by 30% (p < 0.05). The clinic covered costs using Medicare billings and Nationally Weighted Activity Units.

**Discussion:**

The findings suggest this integrated care model was feasible and perceived as beneficial by both patients and providers. The clinic offers a promising model of practice that could be developed further to roll out in other regions for rural delivery of care.

## Introduction

Diabetes is an increasingly prevalent and costly chronic disease, with 643 million cases projected by 2030 worldwide [1]. In Australia, diabetes is one of the leading causes of disease burden [2,3]. The condition has traditionally been managed by specialist services either at hospitals or in private clinics, with limited interaction between hospital endocrinologists and general practitioners [4]. Most diabetes care is delivered by primary care providers in the community [5,6,7]. This fragmentation of care between specialist and primary care services has exacerbated the burden of diabetes and compromised the quality of the care these patients receive [8,9].

In Australia and internationally, integrated care programs aim to address the fragmentation of services that patients with type 2 diabetes experience [10,11]. Integrated care is the provision of seamless, effective and efficient care that responds to all of a person’s health needs across physical, mental, and social health in partnership with the individual, their carer and family [12]. International evidence suggests that integrated care by a multidisciplinary team, including primary and secondary care, can provide consistent management for patients with diabetes and multiple comorbidities [6,13,14]. Integrated diabetes care involving at least two health care providers (an endocrinologist and a GP), can improve clinical outcomes and shared decision-making experience for patients with diabetes [15].

As part of a joint integrated care initiative, Western Sydney Diabetes (WSD) [16] implemented a newly developed model of care called the Mt Druitt Community Diabetes Clinic (MDCDC) located in Mt Druitt Community Health Centre. The Mount Druitt location was selected as this area has a high prevalence of diabetes, with an estimated 29% of adults tested at a General Practice with HbA1c > 6.5% [17]. Adding to this burden, people with diabetes living in Mount Druitt have little access to specialist care compared to other suburbs. Compared to traditional hospital outpatient or ambulatory clinics, the Community Clinic in the Community Health Centre [18] provides treatment and care for people in community settings, away from hospitals. This clinical model has proven non-inferior to routine diabetes clinics in a large cluster-randomized trial, with benefits to routine clinics on key patient metrics such as HbA_1c_ [19]. Despite these potential advantages, the implementation of this model at Mount Druitt has not been evaluated. Common barriers to the implementation of new integrated services include care being shifted to general practices without targeted remuneration for diabetes management, large influx of referrals of non-complex diabetes and insufficient resources to sustain the service [20].

The aim of this study was to test the feasibility of this innovative model of integrated specialist service, to inform local service delivery improvements in Western Sydney Diabetes and future roll-out opportunities.

## Ethics Approval

Ethical approval was obtained from the Western Sydney Local Health District for quantitative (HREC Ref: 2022/ETH00091) and qualitative (2021/ETH00289) components.

## Methods

### Model of care

**Figure 1:**
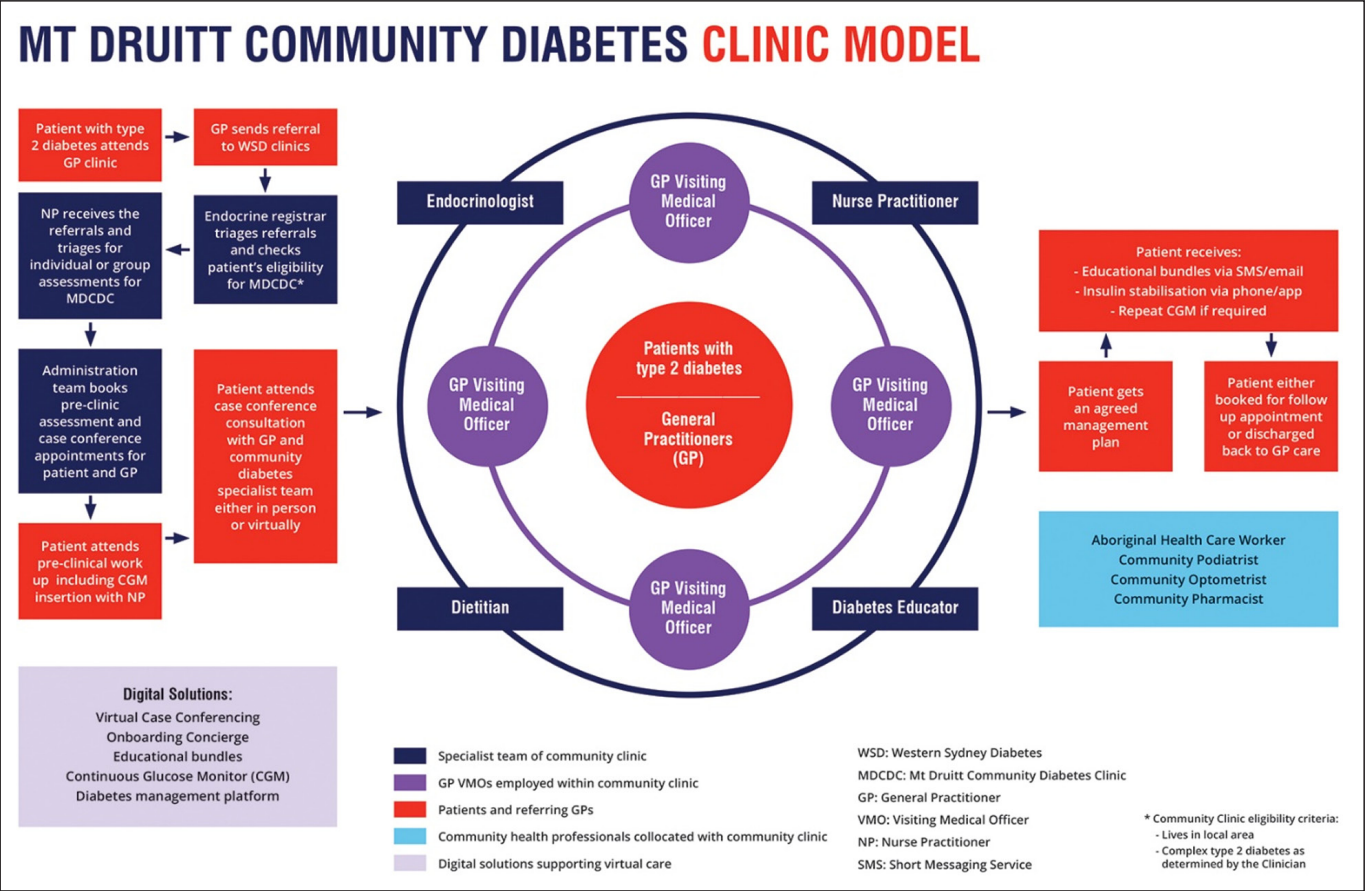
Mt Druitt Community Diabetes Clinic (Integrated care) model.

The new Community Diabetes Clinic model (Figure 1), launched in May 2020, includes a new batch of four General Practitioners employed as Visiting Medical Officers (GP VMO), on 6 monthly appointments. This model has also incorporated diabetes case conferencing [6] as a key foundational aspect of the clinical approach. The GP VMOs conduct diabetes case conferencing with patients, referring or regular GPs and community health providers. An endocrinologist supervises and co-consults with the GP VMOs, so they are upskilled with in-depth specialist approach and apply the skills in their general practice. A full-time nurse practitioner manages the flow of patients booking, pre-clinic consultations including arranging Continuous Glucose Monitoring (CGM), stabilises insulin and management between appointments and provides diabetes education as needed. Patients are referred to the clinic by their GP. Each case is reviewed by the multidisciplinary team – including the four GP VMOs and the supervising endocrinologist from WSD, as well as the nurse practitioner, diabetes educator and dietitian. Patients seen by the GP VMO and other members of the multidisciplinary team as appropriate. The referring GP joins this case conference via telehealth to discuss their patient’s management plan.

### Context

With the onset of the COVID-19 pandemic in 2020, switching from face-to-face consultations or case conferencing to virtual became necessary as people with diabetes have avoided hospitals. In Western Sydney the trend before the pandemic was an increase in the proportion of people with diabetes attending hospitals and other healthcare services, however with the onset of the pandemic this rate dropped dramatically and has since stayed low [21]. Western Sydney Diabetes (WSD), including MDCDC, was an early adopter of Virtual Care (VC) during the COVID-19 pandemic (See Table 1). Since then, MDCDC has been operating a combination of both virtual and in-person modalities.

**Table 1:** Western Sydney Diabetes Virtual Care suite of digital solutions.


INTERVENTION COMPONENT	DESCRIPTION

Diabetes Case Conference (DCC) including specialist team, referring GPs and patients using myVirtualCare platform (myVC) and new ‘concierge’ service.	DCC aims to build referring GPs’ capacity and agreement with the management plan. *myVirtualCare* platform, developed by NSW eHealth and Agency for Clinical Innovation, is a custom-built web-based videoconferencing platform that provides secure virtual consultation room and mimics the physical workflow of a clinical consultation. A new ‘concierge’ service to support patients and GPs to trial the myVC platform and be technically ready in joining the virtual waiting room. The concierge administration team send text messages confirming the appointment booking and the link to myVC for virtual consultations. It also supports onboarding of patients and GPs by testing the myVC link, audio-video settings and connections.

Education bundles	Short educational videos (2 minutes) created by WSD and fact sheets from nationally renowned bodies to enhance self-management for patients with diabetes

Continuous Glucose Monitoring (CGM)	Used for clinic patients to reveal detailed glycaemic profiles over two weeks showing glucose variability (especially hypoglycaemia), evidence of calorie intake and providing insight to patients and providers ways to better use medication and lifestyle changes

Diabetes Management platform	Sharing clinical information for care team and self-management application with patients


### Design

The evaluation included mixed methods to explore the feasibility of implementation of this model, including acceptability to both patients and providers, and improvements to clinical outcomes:

1. Quantitative analysis of patients records who attended the clinics during June 2020 to June 2021.
2. Qualitative study to explore the experiences of patients with type 2 diabetes and healthcare providers involved with the model, including its virtual care component.

### Outcomes

#### Limited Efficacy testing

A retrospective evaluation of the clinic was conducted using routinely collected medical records from the Mount Druitt clinic. This included appointment details, demographic information, pathology results and other biometric data. Key indicators were based on best practice as per American Diabetes Association guidelines, including monitoring (HbA_1c_ target level, eGFR), treatment (medications) and continuity of care [22]. This also included medication use: SGLT2s, GLP-1 RAs, sulfonylureas, metformin and insulin. Data was collected and stored on secure medical records platforms.

We included all patients with appointments made at the clinic between June 2020 and July 2021. Clinical data were collected by the WSD Nurse Practitioner (AM), and compared for patients before their initial appointments and then 3 to 6 months after. All analysis was conducted in Stata 15 using t-tests for continuous variables, and chi-squared tests for categorical variables. Patients without follow up data were excluded from the review. This mostly occurred because patients had not required follow-up (i.e. less than 3 months had passed; 1%) or because they had not yet attended follow-up when this review was conducted (36%).

#### Acceptability

##### Participants

Eligible participants included:

- Adult patients with type 2 diabetes who had attended an appointment at MDCDC via myVC and/or in-person.
- General Practitioners who had referred their patients with diabetes to the MDCDC and attended a joint case conference.
- Staff and health professionals working in the MDCDC and co-located in the community health centre.

##### Recruitment

Participants were informed and invited to participate in the interview by WSD clinicians during appointments and case conference consults. GPs and patients were followed up by SR via phone/email to obtain consent and schedule the interview. Convenience sampling strategy was used to recruit patients and providers who have accessed MDCDC services.

##### Data Collection and analysis

Semi-structured interviews with 10 patients and 5 general practitioners (including 2 VMOs) were completed between May-August 2021, either in person in clinic offices or online. Interview questions (Appendix 1) focussed on benefits, challenges and overall experience. Participants were also asked demographic questions (See Table 2).

**Table 2:** Interview participants characteristics.


PATIENT CHARACTERISTICS (n = 10)	n	%

Gender		

Male	4	40

Female	6	60

Age group		

Less than 29	1	10

30–49	3	30

50–59	2	20

60 and above	4	40

Education		

High school or less	5	50

Certificate or equivalent	2	20

Undergraduate degree or above	3	30

Country of birth other than Australia	4	40

Language spoken other than English	4	40

Indigenous community	1	10

**GP CHARACTERISTICS (n = 5)**		

Gender		

Male	0	0

Female	5	100

Country of birth other than Australia	3	60

Language spoken other than English	3	60

Indigenous community	0	0

Practising more than 10 years in Australia as provider	5	100


Interviews were recorded as audio files and stored in a secure folder within the WSLHD network. Audio files were transcribed verbatim and transcripts were analysed using framework analysis, a matrix-based approach to thematic analysis that involves 5 steps: familiarization with the data, indexing (assigning a code to each line of data), collating similar codes into themes, charting data into a thematic framework, and synthesis and interpretation. The research team included expertise in public health and health literacy (JA, KM, CB), and community specialist service (SR, GFM, RJ). A subset of transcripts was coded by two researchers (SR and JA or CB) and themes were analysed collectively with a continuous process of comparing codes to data and discussion of potential themes.

Two focus groups were also held in July 2021 with 10 clinic staff and 3 co-located allied health personnel in the community health service. Both sessions were held via zoom due to COVID-19 restrictions and facilitated by an independent external consultant (DR). The workshops were recorded on zoom, results analysed by the consultant and synthesised along with overarching themes such as implementation outcomes, patient and provider experiences. Individual participant details were not recorded for the focus groups for anonymity. (Appendix 2: Discussion guide for workshops)

## Results

### Sample size and composition

The clinic was attended by a total of 209 individual clients, providing 3,412 appointments and 2,130 occasions of service during the 2020–21 financial year. This includes new and follow up appointments and mix of in-person and virtual appointments.

**Figure 2:**
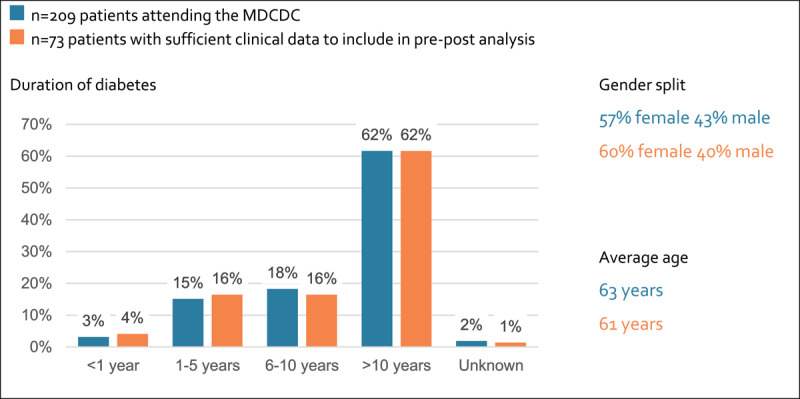
Patient characteristics of total pool and analysis sample.

We analysed 73 patients who had sufficient clinical follow-up data to be included in this pre-post analysis. Demographic characteristics and diabetes history of the sub-sample (n = 73) were close to those of the broader pool (n = 209). See Figure 2.

### Limited efficacy testing

Patients attending the clinic improved their HbA_1c_ levels, dropping from an average of 9.6% to 8.3% (p < 0.0001). A clinically significant reduction in HbA_1c_ is generally considered to be anything greater than 0.5% [23]; here the average decline was 1.3%.

On average, there was no significant difference between pre and post eGFR values (p = 0.32). Of the 54 people with pre and post values for eGFR, 70% either saw an improvement in their values (35%, n = 19) or saw these values hold steady (35%, n = 19). The remaining third (30%, n = 16) saw a decline between their initial and follow-up appointments.

On presentation, 36% of the patients were on SGLT2 inhibitors, with 18% on GLP-1 receptor agonists. After attending the clinic, this lifted to 45% on SGLT2 inhibitors and 49% on GLP-1 receptor agonists. Meanwhile sulfonylurea use dropped from 27% of the population to 5% at follow-up. Insulin use improved, with patients’ average daily dose dropping from 31 units per day to 25 units per day between the initial consultation and the follow-up (p = 0.005). These changes are consistent with better diabetes management.

### Acceptability

Participants included patients (n = 10) and, health providers including referring GPs (n = 5), MDCDC staff (n = 10: GP VMOs, endocrinologist, nurse practitioner, diabetes educator, dietitian) and co-located allied health professionals (n = 3). Interview participant characteristics for patients and referring GPs are summarized in Table 2; these were not collected for focus groups for anonymity.

#### Patients’ experience

Almost all interviewed patients (9/10) used virtual appointments via myVC platform – both audio and video conference. They mostly connected using their phone at home. Half of participants had their GP join the conference.

Participants who attended the clinic either by virtual appointment and/or in-person provided consistently positive feedback about their experience and felt confident in the quality of care and treatment they received through integrated care. However, some difficulties were encountered including limited transport support, internet and communication. Three major themes were generated from the interviews. See Table 3 for supporting quotes.

**Table 3:** Themes from patients’ interviews.


PATIENTS’ PERSPECTIVES	SUPPORTING QUOTES

THEME 1. PERCEIVED BENEFITS AND STRENGTHS OF THE COMMUNITY CLINIC	

Access to multi-disciplinary team care	“just directing me to the right people…Best for teamwork, team for the doctors, team for me” (Pt01).

Convenient location	“I’ve lived in Mount Druitt for 55 years and it’s the best thing that’s ever happened… it’s handy, very, very handy (Pt02).

Virtual care is efficient	“Anything to keep out of the COVID….I’m one of those ones that my immune system, I get attracted to something that I shouldn’t have…”(Pt02)

Concierge service is helpful	“they sent me a link and a time to the appointment. And I think, I think it was like a couple of days they rang, they contacted me on how to use it…they’d go through it step by step with me” (Pt06)

**THEME 2. PERCEIVED CHALLENGES AND LIMITATION OF DELIVERY OF MODALITIES**	

Virtual care limits discussions	“I didn’t speak to the doctor face-to-face, because they take it to another level, who takes everything in, all the reports in. And then she comes back and says, ‘this is what the doctor said, and this is how you’ve got to do it” (Pt02). “face-to-face is better… well, then they can tell me what is wrong with me… then they can tell me what they’re talking about in plain English. Not doctor-doctor English” (Pt07).

In-person appointment challenges	“…I can’t sit in a car for too long…because sometimes I get anxiety…”(Pt02)

Need for technical pre-training	“maybe train the GPs before, because my GP didn’t know how to connect…she had to ring someone to see how to do it. I just thought maybe they should know before starting up on Virtual” (Pt01)

**THEME 3. CONFIDENCE WITH DIABETES MANAGEMENT AFTER ACCESSING THE COMMUNITY CLINIC**	

Opportunity to engage Self-management support	“they would tell me something and then they would ask, you know, what I thought and, did I understand” (Pt06). “I’ve got the information where I need it and also my doctor can, um, access it whenever” (Pt06)


**Theme 1: Perceived patient benefits and strengths of the community clinic**

Participants highlighted many benefits of the community clinic (Table 3). For example, each participant felt positively about having access to integrated care. They emphasised the benefits of working with a team of health professionals from hospital and community settings. Another benefit was that the physical location of the practice within the Community Health Centre instead of hospital itself was considered a convenience. Participants who attended appointments virtually appreciated the minimal wait times, the convenience of attending from home (and reduced COVID-19 risk) and reported that the virtual visits were highly productive as the clinicians were well-prepared. Participants described how support from the concierge services was also useful. For example, participants appreciated receiving SMS messages to confirm appointments, link to virtual platform and conducting technical checks before the appointment.

**Theme 2: Perceived challenges and limitation of delivery modalities**

Participants reported challenges between the two modalities – virtual and in-person appointments (Table 3). For virtual, they felt sorting out technical issues during the appointment cuts the actual consult time and suggested prior training of GPs for virtual connection might preserve timings for consultation. In some instances, patients in virtual consultations were put on hold to allow for internal discussions, for example GP VMOs having brief discussions with the supervising endocrinologist. Although patients understand the process, a few noted that this would have been a different experience for in-person appointment. Participants who have attended both modalities felt virtual consultations were more in doctors’ language and hard to ask questions. For in-person appointments, the main challenges reported were remembering medications and taking all relevant records. Whereas this issue was resolved by virtual appointments due to instant access to records. Another challenge noted was experiencing anxiety about commuting for the appointment.

**Theme 3: Confidence with diabetes management after accessing the community clinic**

Participants felt satisfied and received good ongoing care to manage their diabetes better (Table 3), particularly receiving follow up support and being contacted on multiple occasions to check on them. This reflects the specific role of the dedicated nurse practitioner conducting pre-clinic checks and follow up for ongoing diabetes management. Participants appreciated involving them in shared care planning, with an opportunity and time to talk about their treatment with the consultants.

#### Healthcare Providers experience

Five referring GPs participated in the interviews, including two GPs who had already completed a 6-month appointment as VMO at Mt Druitt Community Clinic.

All participants were female and had been practising for more than 10 years in Australia. Three out of five spoke a language other than English (See Table 1). All of them were aware of the CGM and Insulin stabilisation service; only two were aware of the education bundles available for patients.

In describing their experience of engaging and providing care in the integrated care model, GP VMOs appreciated the strong support from the specialist team, as well as the positive feedback from the patients and referring GPs. Clinic staff consistently agreed that the model facilitated a positive teamwork, enabling the building of strong professional relationships for them. The responses from providers developed three major themes as described below. See Table 4 for supporting quotes.

**Table 4:** Themes from providers interviews and focus groups.


PROVIDERS’ PERSPECTIVES	SUPPORTING QUOTES

THEME 1. PERCEIVED STRENGTHS OF THE MODEL	

Efficient multi-disciplinary team	“A small multidisciplinary team is an effective way to deal with complex cases. We are all around the table and deliver the care instantly. Not reliant on email or mail – no bureaucratic hold ups. The group is small and can deliver all the care in one day.”(Clinic staff)

Strong professional growth in diabetes management	“Because you get the input and also get the education…that’s a really valuable thing for me as well, so that with my other patients with diabetes I’m getting much more comfortable using the newer types of medication and understanding where they fit” (GP02).

Improved continuity of care	“If the patients goes out to see a specialist and then comes back the care’s much more fragmented, so this, this model provides more of the holistic care… and the patient as seen that the dieticians, or diabetes educator or spoken to them individually, but then they can see everyone working as a team. And I think that’s a positive thing for the patient” (GP02).

**THEME 2. PERCEIVED CHALLENGES AND LIMITATION OF DELIVERY OF MODALITIES**	

Difficulty using various virtual platforms and devices	“I did first video conference for one of the patients, I can connect somehow, but it’s so hard, you know? So three-way conversation. Patient couldn’t hear. I couldn’t hear. Maybe you guys have special software” (GP05).

Lack of active participation by referring GP	“With this model we are highly reliant on the referring GP. They usually are not expecting that they need to be present at the time. It’s probably 50–50 between GPs who contribute and those that don’t” (Clinic staff).

Limited ability to contact patients	“Our client base is not really tech savvy. You have to call them, and they won’t answer private or blocked numbers. Texting first can really help. Rapport building is key” (Clinic staff).

**THEME 3. PERCEIVED AREAS OF IMPROVEMENT/REQUESTED IMPROVEMENTS**	

Simple and easy to understand educational resources	“The NDSS [National Diabetes Services Scheme] resources are very wordy. I use other ones – Bakers Institute, or ones from Queensland, or I’ve developed my own. Diabetes Australia have good resources. You need things that are visual rather than simple. There are some good exercise resources too” (Clinic staff).

More digital monitors	“If we could have more of those [continuous] glucose monitors that would be great. They’re a really effective educational tool, raising awareness to trigger self-management and conversations about self-management. Otherwise, this cohort is pretty bad at doing the finger prick thing” (Clinic staff).


**Theme 1: Perceived strengths of the model**

All participants mentioned the efficiency of a multidisciplinary team working together and organised communication between clerical and clinical staff. A particular strength noted was effective patient care that is timely and well-coordinated. For referring GPs and GP VMOs working in the clinic, key benefits highlighted were skills and knowledge gained from direct access to specialist expertise, quality of the information exchanged and the efficiency of multi-disciplinary learnings received during the pre-clinic and joint consults. Referring GPs felt being part of the team, with a central role, allowed much better continuity of care rather than waiting for a letter from the specialist clinic advising the treatment plan. Providers were impressed by the way the model allowed patient-centred and coordinated diabetes care in one visit.

**Theme 2: Perceived issues with the model**

There were a few challenges raised, particularly about huge variations in the type and age of devices used across the GP practices and their network or need to set up for virtual connections. These issues required a lot of technical troubleshooting. Further challenges included referring GPs’ variable engagement with case conferencing and the expectations with scheduling modifications to work around other appointments in their practice. From providers’ perceptions a few inherent challenges of their vulnerable clients were identified, including limited access to transport and mobile data or home internet. This affected their capacity to attend in-person appointments, limited use of virtual appointments and digital resources (such as education bundles and CGM technology), and meant they were less familiar with the required technology.

**Theme 3: Perceived areas of improvement/requested improvements**

Participants perceived that various promotional and educational resources designed for patients were not user friendly and suggested they need to be very simple and to meet their limited health literacy levels, including the education bundles. Scanning of the CGM sensor requires patients to either use their mobile phone (requiring internet) or via a reader. However, with limited number of readers available, providers recommended that a larger supply of readers would maximise the benefits of the technology for the patient group.

## Discussion

The key findings from this mixed-methods evaluation of the new Community Diabetes Clinic indicate that a real-world implementation of the model resulted in patient benefits and reported improvements across patient and provider experience and better health outcomes. The study demonstrates the feasibility of the model in terms of limited efficacy testing, and acceptability [24].

The clinical outcomes for patients indicated by pre-post data showed a significant and clinically meaningful reduction in the average HbA_1c_ from baseline and reflects the average HbA_1c_ achieved in the diabetes case conferences across other WSD clinics [6]. Optimisation of medicine was achieved as the use of newer medications, SGLT2 inhibitors and GLP-1 receptor agonists, have increased among clinic patients after attending the clinic. The lack of significant difference in pre-post eGFR levels is a positive result, as normally when diabetes is poorly managed eGFR values will decline over time.

The model received strong support from the clinicians who were involved in its delivery, as well as a high referral rate and consistently positive feedback from referring GPs. The model also built the value of insulin stabilisation as an effective treatment approach and use of CGM to learn how patients’ lifestyle impact their health. All patients reported that virtual care improved their confidence in self-management and many also preferred virtual care. These findings add further evidence to the acceptability of telehealth, especially under lock down restrictions of the COVID-19 pandemic, in line with previous research [25].

However, there were some barriers to the model. The education bundles appeared to have low uptake amongst both patients and providers. Our qualitative findings indicate there may have been a gap in promoting these resources or limited dissemination, which led to low awareness. Another implementation barrier is the technical issues for GPs uptake on virtual care. This mostly relies on the difference in the practices’ infrastructure of devices and network.

Evaluation outcomes from this study contributed to a business case for securing permanent funding and resources for running this model as business as usual. This addresses the financial barriers to integrated care raised in the broader literature [26], which was not raised as a main issue by participants. Further, this model also addressed another key barrier of accessing appropriate remuneration from Medicare Billing Scheme for participating in joint case conferencing.

The adoption of virtual care has helped with the success of the clinic as it was launched at the challenging times of the pandemic. As indicated by various studies, virtual care has been effective in the management of type 2 diabetes, especially those interventions involving self-management strategies [27]. As the public health restrictions of the COVID-19 pandemic have been relaxed, the model has been operating with a hybrid approach including both in-person and virtual modalities. Moving forward, it will be important to ensure that the model can overcome technological barriers such as the limited availability of devices and internet access, and provider and patient IT skills.

Our evaluation has demonstrated the feasibility of an integrated care model in practice. While a similar model has previously shown benefit in a pragmatic clinical trial [19], this is to our knowledge the first real-world evaluation showing that the model can work in another setting. Video case conferencing, telehealth concierge and dedicated nurse practitioner are the unique key features that helped with implementation and has proven feasibility of this model. It is often challenging to implement integrated care models in clinical practice [28,29] as the populations they serve often have complex needs, and come from diverse groups, and these kinds of interventions may face many organizational barriers. This project fits into a broader category of integrated care that joins primary and secondary healthcare services, and connects with the international experience that providing integration is, in practice, possible despite structural barriers [30].

### Strengths and Limitations

The success of this model is notable given the challenging conditions when it was launched during the COVID-19 pandemic. The evaluation using both quantitative and qualitative methods has revealed that this community-oriented model could feasibly be replicated in a range of different clinical contexts and geographical locations. The clinical data analysis does not compare client outcomes with those of a control group. This is partly due to difficulties in identifying an appropriate comparator group for patients accessing ‘usual care’, but also because on an Australian clinical trial has already shown that patients receiving integrated clinical care for diabetes experience similar or better outcomes than patients receiving usual care. The participants recruited to the qualitative analysis were those who accessed this service and volunteered to be interviewed. Therefore, we acknowledge there is a high risk of positive bias. Further the evaluation has no access to feedback from non-attending patients about their reasons for not following through on the referral or for cancelling their appointment. However, we note that the cancellation rate for the MDCDC is lower than the cancellation rate for the usual complex diabetes clinics in Blacktown hospital. A further limitation to this study is finding out reasons why referring GPs did not engage with joint case conference or involve as part of care team.

Importantly, this paper was conducted as a pragmatic evaluation of the service and did not aim to demonstrate a causal connection between the service and outcomes. Given the previous work in which this model has been proven to have similar outcomes to traditional clinics (14), we aimed to review key implementation questions as well as patient and provider perspectives. In particular, without a control group and random prospective assignment, it is impossible to know how much of the benefit seen in the patients reviewed at this clinic is directly related to the clinic itself. However, while this is a limitation to the interpretation of our results, the study suggests that the findings of previous RCTs can be effectively put into practice in a different clinical setting such as the MDCDC.

**Lessons learnt:**

✓ *Clinical improvements demonstrated in a different RCT setting were replicated in pre-post analyses in this case study*.
✓ *The integrated care model was acceptable to both patients and providers in this study*.
✓ *Some barriers to technology and access remain, requiring flexibility in delivery format*.

## Conclusion

Integrated care models such as this Community Diabetes Clinic can be feasible when enabled by virtual care. This case study demonstrated a promising integrated model that was acceptable to both health providers and patient with improved clinical outcomes. Future research could investigate the feasibility of adapting this integrated model in other clinical settings and evaluation of the cost-effectiveness.

## Additional Files

The additional files for this article can be found as follows:

10.5334/ijic.7548.s1 Appendix 1. Interview guide for patients.

10.5334/ijic.7548.s2 Appendix 2. Discussion guide for workshops.
