# Supplementary material for: Qualitative and Quantitative Evaluation of an Innovative Primary and Secondary Diabetes Clinic in Western Sydney
Source: Int J Integr Care. 2024 Feb 21;24(1):13. doi: 10.5334/ijic.7548 (PMC10885848; doi:10.5334/ijic.7548)
Supplement: Appendix 1. — Interview guide for patients. [file ijic-24-1-7548-s1.pdf]

APPENDIX 1

Interview guide for patients

Introduce self to the participant and if anyone else present to each other.

Suggest they might want to have a glass of water available while they do the interview, while you set up.

Reiterate the participant information sheet:

Introduction: *Thank you for agreeing to be interviewed. We are doing an interview study to explore patients' experiences, both good and bad, of using Western Sydney Diabetes Virtual Clinic (WSDVC) for diabetes attending Mt Druitt Community Diabetes clinic. We need your feedback to help improve WSDVC, Mt Druitt Clinic services, GPs' experiences and patients' healthcare and experiences in western Sydney.*

Confidentiality/recording: *Everything you say will be strictly confidential. Your name will be removed from the written transcript, which will be made from an audio recording of the interview. Any identifying names will be removed from the transcript. The interview includes looking at the WSDVC (myVirtualCare platform and explain as telehealth/AV conference) and Community diabetes clinic model.*

The Interview: *The interview will take approximately 45 minutes. If you'd prefer not to answer any of the questions just let me know and we'll move on to the next question. You can also stop the interview at any time. The interviews will be recorded using a voice recorder and transferred to a secure electronic storage folder in WSLHD drive.*

Does that sound ok? Do you have any questions before we start?

Consent: *If you are happy to start could you please sign the consent form (if it hasn't been signed before).*

We have been using a virtual care platform for joint specialist, GP and patient consultations since the beginning of the pandemic in 2020. The new Community Diabetes Clinic model was established in Mt Druitt Community Health Centre in May 2020, which was precisely during the peak pandemic. This new model embarked fully on to virtual care. Therefore, it is very important that this platform / virtual care solutions are integrated well with your GP, healthcare team and yourself, as everyone plays an important role in your diabetes care. This interview is focussed on hearing from a patient's perspective and experiences and how we can continue to adapt, improve/implement the virtual care in Western Sydney.

<u>*START RECORDER***</u>**

Part 1: Virtual Care Appointment

1. Is this your first or follow up appointment?
2. What was the wait time to get an appointment booked?
3. Were you able to get an appointment time that suited you?

   Yes / No / didn't have an appointment booked in advance (walk in?)
4. How many times have you been contacted by the booking/online support team?
5. Who did you see during your virtual care appointment?

   ☐ Specialist
   ☐ Doctor
   ☐ Diabetes educator

- ☐ Dietitian
- ☐ Podiatrist
- ☐ Mental Health Professional
- ☐ Psychologist
- ☐ Other healthcare professional(s)

6. What type of virtual care method did you use at your appointment?

   - ☐ myVirtualCare – audio & video
   - ☐ myVirtualCare – audio only
   - ☐ Telephone only – mobile or landline
   - ☐ Other

7. Did you experience any problems with the connection or technology during this appointment?

   Yes or No

8. Did you receive technical support from our online/technical support (concierge) team to help you participate in your virtual care appointment?

   Yes / No / don't know or can't remember

9. Were you adequately prepared for this appointment – technically as well as having records/results/sugar readings etc?

   Yes / to some extent / No

10. Did your GP join this appointment? If yes did you join this appointment together with your GP?

Part 2: Care and treatment

11. WSD has extended their services with opening a new community diabetes clinic in Mt Druitt. How do you think this new service with virtual care is helping your care and treatment?

12. Do you see this new model of community diabetes clinic as a combined approach for your care and treatment?

    Do you see any problems with this approach?

13. Were you involved as much as you wanted to be in decisions about your care and treatment?

14. Do you think your privacy was maintained during/before/after your virtual care appointment?

15. WSD provides/sends education bundles (videos and fact sheets) to patients phone or email for ongoing education.

    a. Did you get any of the bundles?

    b. Have you seen any of the bundles?

    c. Do you have any suggestions to make the contents better?

16. Many patients were offered a subsidised continuous glucose monitor (CGM) to improve their blood glucose profile.

    a. Are you aware of this?

    b. Did you learn from this?

    c. How could we make this better?

Part 3: Overall experience

17. Did the care and treatment received through virtual care help you?

18. How did you experience with virtual care with what you are used to going to the doctor before COVID?

    What aspects did you like more?

    What did you like less?

    Would you use virtual care again?

19. Thinking about your experiences

    a. In terms of virtual care, what have been:

        – the benefits for you?

        – the challenges for you?

    b. In terms of the new community clinic in Mt Druitt, what have been:

        – the benefits for you?

        – challenges for you?

20. Do you have any additional feedback regarding VC?

21. Do you have any suggestions for additional services for people with diabetes that could be integrated with this service?

Part 4: About You

*I would like to finish this interview by asking a few general questions about you. This will be used to describe the whole participant group, and will not be recorded with your interview [**turn recorder off**].*

22. What year were you born?

23. In which country were you born?

    If not Australia: In what year did you move to Australia to live?

24. What is your gender?

    ☐ Male
    ☐ Female
    ☐ Other

25. What is the highest level of education you have completed?

26. Are you of Aboriginal and/or Torres Strait Islander origin?

    ☐ No
    ☐ Yes – please specify:_______

27. Do you speak another language at home other than English?

    ☐ No
    ☐ Yes – please specify:_______

Interview guide for providers

Introduce self to the participant and if anyone else present to each other.

Suggest they might want to have a glass of water available while they do the interview, while you set up.

Reiterate the participant information sheet:

Introduction: *Thank you for agreeing to be interviewed. We are doing an interview study to explore providers' in particular GPs' experiences, both positive and negative, of using WSDVC in managing patients with type 2 diabetes. This study is for those of your patients who have recently attended the new Community Diabetes Clinic in Mt Druitt Community Health Centre. We need your feedback to help improve WSDVC, Mt Druitt Community Clinic services and patients' healthcare and experiences in western Sydney.*

Confidentiality/recording: *Everything you say will be strictly confidential. Your name will be removed from the written transcript, which will be made from an audio recording of the interview. Any identifying names will be removed from the transcript. The interview includes looking at the myVirtualCare platform and community diabetes clinic model.*

The Interview: *The interview will last approximately 30 minutes. If you'd prefer not to answer any of the questions just let me know and we'll move on to the next question. You can also stop the interview at any time. The interviews will be recorded using a voice recorder and transferred to a secure electronic storage folder in WSLHD drive.*

Does that sound ok? Do you have any questions before we start?

Consent: *If you are happy to start could you please sign the consent form (if hasn't been signed before).*

We have been using a myVirtualCare platform for joint specialist, GP and patient consultations since the beginning of the pandemic in 2020. The new Community Diabetes Clinic model was established in Mt Druitt Community Health Centre in May 2020, which was precisely during the peak pandemic. It is very important that this platform and virtual care solutions are integrated well with the patient's GP, the GP VMOs and specialist team of WSD as this new model of integrated approach plays an important role in diabetes care. This interview is focussed on hearing from a GP's perspective and experiences and how we can continue to adapt, improve/implement the VC and community diabetes model in Western Sydney.

*****START RECORDER*****

Part 1. Information resources and infrastructure

1. What do you already know or what information have you received about telehealth or WSD Virtual Care for appointments with WSD?

   (Prompt for WSD telehealth brochure, websites, OST phone / email services).

2. What types of equipment/devices do you have to use for virtual care appointment?
3. Did you experience connection or technical problems for VC appointments?
4. Does your practice support video conferencing or only telephone conferencing with the patient or the specialist?

   (prompts: your practice embrace this new video technology or feel that it was just too hard to use at the time COVID had so many other demands)

Part 2: Concierge support and experience

5. Were you aware that WSD has a 'concierge' service to assist the patient and the practice to join the WSD waiting room and be ready to join the consultation with the WSD team?

6. How did you find the myVirtualCare training and support provided to you and your practice staff by the WSD ‘concierge” service?

   (prompt/follow up) What aspects of support helped or hindered the implementation?

7. Was the process efficient and timely or was there a large amount of time lost in getting the technology to connect and for everyone who needed to be able to come together efficiently?

8. 30 minutes has been allocated for the joint consultation (specialist, patients and GP) on the platform.

   Was this time about right? Too long or too short?

9. The Specialist team spent time talking with you and also talking with the patients. Was that the right approach or would you like more or less time to talk with the specialist team?

10. Were you able to bill Medicare for the online consultation?

Part 3: Care and experience

11. Do you think your patients had a positive experience of care through the WSDVC clinic?

12. WSD has adapted to virtual care and has extended their services with opening a community diabetes clinic in Mt Druitt. This model has GP VMOs and specialist team having a joint consult with you and your patient in the same appointment to discuss the care plan.

    - What do you feel or think about this new model of joining the consultation with VMOs?
    - Is it helping you to deliver care efficiently?

13. One of the aims of the virtual care is to save the practitioner and patients time and effort where possible whilst maintaining a high quality of care.

    - Do you see this as a useful feature?
    - What problems/risks do you foresee with this approach?
    - Did this create a problem with time management at the practice to make this feasible?
    - How could this be improved?

14. Joint consultations has two aspects, one is for WSD Specialist team to jointly manage the patient with you and second is to provide information to you so you could better manage all your patients with diabetes.

    - Was this balanced approach working/worked well for you?
    - Was it the right balance?
    - How could we make it better?

15. Some patients may have been sent education bundles (videos and fact sheets) to their phone or email for ongoing education.

    - Are you aware of these bundles?
    - Did you see any of the bundles?
    - How could we make the contents of the education bundles better?
    - How could we use or make the dissemination of the education bundles better?

16. Many patients were offered a subsidised Flash Glucose Monitor (CGM) to improve their glycaemic profile.

    - Are you aware of this?
    - Did the patient learn from this?
    - Did you learn from this?

- How could we make this process better?

17. Our Educator provides insulin stabilisation of Blood Glucose Level service for several weeks for certain patients.
    - Are you aware if any of your patients use/used this service?
    - Do you have any suggestions to make this service better?
18. Do you have any additional feedback regarding virtual care?
19. Do you think any other service that could be integrated with this service for management of your patients with diabetes?

Part 4: Additional questions

*I would like to finish this interview by asking a few general questions about you. This will be used to describe the whole participant group and will not be recorded with your interview [**turn recorder off**].*

20. What year did you qualify as a doctor?
21. What is your medical specialty?
22. What is your gender?
    - ☐ Male
    - ☐ Female
    - ☐ Other
23. In which country were you born? ___________________

    If not Australia: In what year did you move to Australia to live?
24. Are you of Aboriginal and/or Torres Strait Islander origin?
    - ☐ No
    - ☐ Yes (please specify)
25. Do you speak another language at home other than English?
    - ☐ No
    - ☐ Yes (please specify)
