# Supplementary material for: Qualitative and Quantitative Evaluation of an Innovative Primary and Secondary Diabetes Clinic in Western Sydney
Source: Int J Integr Care. 2024 Feb 21;24(1):13. doi: 10.5334/ijic.7548 (PMC10885848; doi:10.5334/ijic.7548)
Supplement: Appendix 2. — Discussion guide for workshops. [file ijic-24-1-7548-s2.pdf]

APPENDIX 2

Discussion guide for workshops

Purpose: The primary purpose of the evaluation is to inform local service delivery improvements in WSD. It is also intended that the evaluation will assist ACI and NSW Health to inform the potential roll-out of future virtual models of care elsewhere in NSW.

Place of workshop in evaluation process

- Qualitative interviews with clinic staff, referring GPs and patients
- Analysis of clinical data
- Analysis of financial data
- Data workshop
- Workshop to test findings

Timing: Strict one hour window so we will race through

Zoom suggestions: Put comments in chat

Recording: Confirm group is happy for the session to be recorded for note-taking purposes.

1. Positives and benefits of Mt Druitt Community Diabetes Clinic

- What worked well for you as clinicians here?
- What benefits are there for referring GPs?
- What benefits are there for patients?

2. Challenges and areas for improvement

- What challenges were there for you as clinicians here?
- How can the clinic increase GP awareness, engagement and capacity?
- What can the clinic do better for patients in future?
- What other multidisciplinary services might be useful in future?
- Are there patient groups who may have missed out?

3. Specific issues – prompt as necessary

- Uptake of multidisciplinary services
- Awareness/uptake/engagement of referring GPs
- Capacity building among referring GPs
- Tech/IT issues
  - Concierge service
  - MyVirtualCare platform
  - Billing
- Virtual care bundle
  - Educational resources
  - Joint specialist/GP case conference service
  - Continuous home glucose monitor
  - Healthy living advice

4. Other aspects of implementation or ideas for improvement

5. Lessons for scaling and virtual care

6. Final comments
